# Long-term follow-up of a combined rituximab and cyclophosphamide regimen in renal anti-neutrophil cytoplasm antibody-associated vasculitis

**DOI:** 10.1093/ndt/gfx378

**Published:** 2018-02-14

**Authors:** Stephen P McAdoo, Nicholas Medjeral-Thomas, Seerapani Gopaluni, Anisha Tanna, Nicholas Mansfield, Jack Galliford, Megan Griffith, Jeremy Levy, Thomas D Cairns, David Jayne, Alan D Salama, Charles D Pusey

**Affiliations:** 1 Renal and Vascular Inflammation Section, Department of Medicine, Imperial College London, London, UK; 2 Vasculitis Clinic, Imperial College Healthcare NHS Trust, London, UK; 3 Department of Medicine, University of Cambridge, Cambridge, UK; 4 Centre for Nephrology, University College London, London, UK

**Keywords:** ANCA-associated vasculitis, cyclophosphamide, granulomatosis with polyangiitis, microscopic polyangiitis, rituximab

## Abstract

**Background:**

Current guidelines advise that rituximab or cyclophosphamide should be used for the treatment of organ-threatening disease in anti-neutrophil cytoplasm antibody (ANCA)-associated vasculitis (AAV), although few studies have examined the efficacy and safety of these agents in combination.

**Methods:**

We conducted a single-centre cohort study of 66 patients treated with a combination of oral corticosteroids, rituximab and low-dose pulsed intravenous cyclophosphamide followed by a maintenance regimen of azathioprine and tapered steroid for the treatment of biopsy-proven renal involvement in AAV. Patients were followed for a median of 56 months. Case–control analysis with 198 propensity-matched cases from European Vasculitis Study Group (EUVAS) trials compared long-term differences in relapse-free, renal and patient survival.

**Results:**

At entry, the median Birmingham Vasculitis Activity Score (BVAS) was 19 and estimated glomerular filtration rate was 25 mL/min. Cumulative doses of rituximab, cyclophosphamide and corticosteroids were 2, 3 and 4.2 g, respectively, at 6 months. A total of 94% of patients achieved disease remission by 6 months (BVAS < 0) and patient and renal survival were 84 and 95%, respectively, at 5 years. A total of 84% achieved ANCA-negative status and 57% remained B cell deplete at 2 years, which was associated with low rates of major relapse (15% at 5 years). The serious infection rate during long-term follow-up was 1.24 per 10 patient-years. Treatment with this regimen was associated with a reduced risk of death {hazard ratio [HR] 0.29 [95% confidence interval (CI) 0.125–0.675], P = 0.004}, progression to end-stage renal disease (ESRD) [HR 0.20 (95% CI 0.06–0.65), P = 0.007] and relapse [HR 0.49 (95% CI 0.25–0.97), P = 0.04] compared with propensity-matched patients enrolled in EUVAS trials.

**Conclusions:**

This regimen is potentially superior to current standards of care, and controlled studies are warranted to establish the utility of combination drug approaches in the treatment of AAV.

## INTRODUCTION

The past decade has seen B-cell depletion therapy using rituximab become an established treatment strategy in anti-neutrophil cytoplasm antibody (ANCA)-associated vasculitis (AAV), both for remission induction and remission maintenance. Its use was initially described as add-on therapy in cases refractory to conventional treatment [1–3], based on experience in other autoimmune diseases. In 2010, two landmark randomized controlled trials, the Rituximab for ANCA-associated Vasculitis (RAVE) and Randomised Trial of Rituximab versus Cyclophosphamide for ANCA-associated Renal Vasculitis (RITUXVAS) studies [4, 5], suggested that it was non-inferior to treatment with cyclophosphamide for induction treatment in AAV. It was subsequently approved for this indication in 2011. Recent cohort studies and one randomized controlled trial—MAINtenance of remission using RITuximab in Systemic ANca-associated vasculitis (MAINRITSAN)—suggest that continued re-treatment with rituximab is also effective in preventing relapse [6, 7].

Current international guidelines suggest that rituximab may be considered an effective alternative to cyclophosphamide in patients presenting with organ- or life-threatening disease, including those with renal involvement [8, 9]. Controlled evidence examining the role of rituximab/corticosteroid regimens in patients with severe renal involvement in AAV, however, is lacking and few studies have examined the combined use of cyclophosphamide and rituximab. Retrospective analysis of a large cohort of patients enrolled in the Glomerular Disease Collaborative Network suggested that combined or sequential use of cyclophosphamide and rituximab is safe and associated with a longer time to relapse compared with rituximab treatment alone, although the case mix in this study was heterogeneous and treatment regimens non-standardized [10]. Prolonged remission after an oral cyclophosphamide and rituximab–based induction regimen has been reported in a subset of patients who also received maintenance therapy with scheduled rituximab redosing [6, 11], providing further evidence that a combination approach is safe and may be associated with a decreased risk of relapse.

Since 2006 we have used the combination of rituximab and low-dose intravenous cyclophosphamide, with oral corticosteroids, for remission induction in renal AAV. Our adoption of this protocol predates the reports of RAVE and RITUXVAS and our initial rationale was to use rituximab to allow a reduction in cumulative exposure to cyclophosphamide, an agent proven to be effective in early disease control. In 2011 we published our initial experience using this strategy (‘CycLowVas’) in a cohort of 23 patients, in whom we observed high rates of remission and prolonged relapse-free survival [12]. We now report our extended experience using this regimen in a larger cohort with long-term follow-up and compare outcomes with patients treated with cyclophosphamide-based regimens in European Vasculitis Study Group (EUVAS) trials. We confirm high rates of clinical response and prolonged B-cell depletion that was associated with low relapse rates and improved patient and renal survival during long-term follow-up.

## MATERIALS AND METHODS

This is a cohort study of patients with renal AAV treated since 2006 at the Hammersmith Hospital, London, UK, using our previously published combination regimen [12]. This report includes 22 patients described in our initial report, with an extended period of follow-up, and an additional 44 cases who have been treated since.

All consecutive patients were considered for treatment with this protocol. Inclusion criteria were active AAV with renal involvement, as defined by the presence of circulating ANCA detected by indirect immunofluorescence (IIF) or antigen-specific assay and either (i) biopsy-proven pauci-immune glomerulonephritis or (ii) active urinary sediment and abnormal renal function in patients with evidence of glomerulonephritis on former biopsy. Patients were assigned to myeloperoxidase (MPO)-ANCA or proteinase 3 (PR3)-ANCA groups according to antigen-specific testing. Two patients negative by antigen-specific assay were positive for perinuclear ANCA by IIF, without granulomatous features, and so were assigned to the MPO-ANCA group.

Exclusion criteria included severe renal disease (serum creatinine >500 μmol/L or requirement for dialysis at initial presentation), diffuse alveolar haemorrhage, cerebral vasculitis or other extrarenal manifestations requiring the addition of plasma exchange, and <1-year follow-up. Patients with contra-indications to cytotoxic therapy (malignancy, acute infection and chronic suppurative lung disease) were not included. Patients were followed up until the last clinical visit prior to 31 December 2015.

The treatment protocol is summarized in Table 1. In brief, the regimen combines two 1 g doses of rituximab, six intravenous doses of cyclophosphamide 500–750 mg and an initial oral prednisolone dose of 1 mg/kg (maximum 60 mg) daily that is rapidly tapered by Month 6 to 10 mg/day maximum. In patients who received intravenous corticosteroids prior to referral to our centre, a reduced initial oral corticosteroid dose was permitted at the physician’s discretion. Corticosteroid taper after 6 months was not protocolized and likewise at the physician’s discretion. Maintenance immunosuppression was commenced at 3 months (or earlier in patients who received shorter courses of cytotoxic therapy) and recommended to continue for at least 2 years.

**Table 1:** Treatment protocol

	Agent	Dose
Cytotoxic therapy		
Day 0	Rituximab	1 g
	Cyclophosphamide i.v.	10 mg/kg (maximum 750 mg)
Week 2	Rituximab	1 g
	Cyclophosphamide i.v.	10 mg/kg (max. 750 mg)
Weeks 4, 6, 8 and 10	Cyclophosphamide i.v.	500 mg × 4
Corticosteroid taper		
Week 1	Oral prednisolone	1 mg/kg/day (maximum 60 mg)
Week 2	25% reduction	45 mg
Week 3	33% reduction	30 mg
Week 4	33% reduction	20 mg
Week 6	25% reduction	15 mg
Week 12		Minimum 12.5 mg
Week 20		10 mg
Maintenance therapy		
From Week 12	Azathioprine	1–2 mg/kg/day (adjusted for TPMT levels)
	Mycophenolate mofetil (if intolerant)	1–2 g/day (targeted to trough levels of 1.2–2.4 mg/L)
Prophylactic therapy		
PJP prophylaxis	Co-trimoxazole 480 mg/day	
	Pentamidine nebulizer 300 mg/month (if intolerant)	
Peptic ulcer prophylaxis	Proton-pump inhibition	
Bone prophylaxis	Vitamin D and calcium supplementation	
Latent TB prophylaxis (in those from high-risk areas)	Isoniazid 150 mg/day and pyridoxine 50 mg/week	

i.v., intravenous; TPMT, thiopurine methyltransferase enzyme activity; PJP, *Pneumocystis jiroveci* pneumonia; TB, tuberculosis.

Disease activity was scored using version 3 of the Birmingham Vasculitis Activity Score (BVAS) [13]. Remission was defined as a BVAS score of 0. At 6 months, patients with persistent urinary abnormalities in the presence of improving or stable excretory renal function and no extrarenal disease activity were determined to have a BVAS score of 0. Renal biopsies were categorized according to the Berden classification [14]. B-cell counts were determined by flow cytometry for CD19^+^ cells at 3- to 6-month intervals and a threshold of 10 cells/μL was used to define B-cell depletion/repopulation. ANCA was detected by IIF (Inova Diagnostics, San Diego, CA, USA) or antigen specific assay (2006–2013: FIDIS Multiplex, Theradiag, Marne-la-Vallee, France; 2013–2015: Immunocap250 CMIA, ThermoFisher Scientific, Waltham, MA, USA). The glomerular filtration rate (GFR) was estimated using the Modification of Diet in Renal Disease calculation [15]. Relapse was defined by an increase in disease activity requiring augmented treatment. Major relapse included patients with recurrent glomerulonephritis or respiratory tract involvement that required re-treatment with cytotoxic agents. Minor relapses included constitutional symptoms, arthralgia and ear, nose and throat (ENT) symptoms that required a reintroduction or increase in dose of oral corticosteroids/immunosuppression.

For unadjusted analysis, all data were regarded as non-parametric and comparison between groups was by Fisher’s exact test for categorical data and Mann–Whitney test for continuous variables. Survival functions and time-to-event analyses were plotted as Kaplan–Meier curves and groups were compared by log-rank test. ESRD- and relapse-free functions were censored for death. B-cell count and ANCA levels were censored at the point of re-treatment with cytotoxic therapy. Graphs were constructed and statistical analysis performed using Prism 7.0 (GraphPad Software, La Jolla, CA, USA).

For case–control analysis, patients enrolled in previous EUVAS trials [Cyclophosphamide vs Azathioprine during Remission of Systemic Vasculitis (CYCAZAREM) [16], Cyclophosphamide in Systemic Vasculitis (CYCLOPS) [17] and Plasma Exchange for Renal Vasculitis (MEPEX) [18]] were identified in a ratio of 3:1 with our patient cohort and propensity matched by age, baseline chronic kidney disease (CKD) staging criteria and ANCA specificity. Cox proportional regression analysis was used to ascertain proportional hazard ratios (HRs) for factors associated with categorical outcomes (death, progression to ESRD, relapse). Covariates included age, sex, ANCA specificity, entry eGFR, entry BVAS and dose of cyclophosphamide. Analysis was performed using SPSS version 23 (IBM, Armonk, NY, USA).

This was a retrospective review meeting the criteria for a service evaluation study and hence did not require approval from a research ethics committee. All patients gave their consent for treatment and received standard care according to our accepted unit protocols.

## RESULTS

### Case identification

Between 2006 and 2014, 184 consecutive patients were treated for new or relapsing renal AAV at our centre, all of whom were considered for treatment with this protocol. Excluded from this analysis are patients who had severe disease manifestations requiring the addition of plasma exchange (*n* = 49), those with ‘double-positive’ ANCA and anti-glomerular basement membrane disease (*n* = 12), those who had contraindications or declined to consent to a component of therapy (*n* = 25) and those who were recruited to clinical trials or other cohort studies (*n* = 30). Of the 68 patients treated, two moved out of the centre in the first year of follow-up. The demographic features of the remaining 66 patients with at least 1-year follow-up included in this analysis are summarized in Table 2. The median duration of follow-up for the entire cohort was 56 months [range 1–120].

**Table 2:** Demographic features and disease status at presentation

	All	MPO-ANCA	PR3-ANCA	P-value
Demographics				
* n* (%)	66 (100)	33 (50)	33 (50)	
Male:female, %	58:42	52:48	64:36	0.46
Age (years), median (range)	62 (17–84)	68 (17–84)	59 (19–81)	0.04
Comorbidities, %				
Respiratory	21	30	12	0.13
Cardiac	12	15	9	0.71
Diabetes	12	15	9	0.71
Hypothyroidism	18	18	18	1.00
Disease status				
* De novo* presentation, %	89	91	88	
BVAS, median (range)	18.5 (12–31)	17 (12–31)	21 (12–29)	<0.01
Creatinine (μmol/L), median (range)	205 (63–479)	213 (74–479)	180 (63–440)	0.56
eGFR (mL/min), median (range)	25 (8–86)	24 (8–71)	33 (8–90)	0.44
Biopsy class, %				0.65
Focal	24	21	27	
Crescentic	33	30	33	
Mixed	42	48	36	
Tubular atrophy, % (range)	10 (0–50)	20 (10–50)	10 (0–40)	0.01

### Baseline disease features

The majority of patients [59/66 (89%)] were treated for *de novo* presentations. All had renal involvement (median creatinine 205 μmol/L, eGFR 25 mL/min; biopsy proven in all but two cases). A spectrum of histological class of disease was observed, although none had sclerotic-class disease. Extrarenal manifestations were present in 59/66 (89%; median BVAS at presentation 18.5). PR3-ANCA patients [33/66 (50%)] were younger and had more extrarenal manifestations and higher BVAS, with less evidence of chronic damage on biopsy than MPO-ANCA patients, in keeping with previously reported clinicopathologic differences between these groups (Table 2).

### Treatment received

All patients received rituximab 2 × 1 g. The median cumulative dose of delivered cyclophosphamide was 3 g (range 1–5.5). Thirteen patients (20%) received fewer than the protocolized six doses of cyclophosphamide due to leucopenia, infection or (in relapsing patents) concerns regarding prior cumulative dose. One non-responding patient (discussed further below) had an additional 4 × 500 mg doses until remission was achieved. Fourteen patients (22%) received pulsed intravenous methylprednisolone prior to referral. The median cumulative dose of total oral and intravenous corticosteroids at 6 months was 4.2 g (range 1.0–6.5).

At 6 months, 58/64 surviving patients (91%) were receiving maintenance immunosuppression: 45/64 (70%) with azathioprine and 12/64 (19%) with mycophenolate mofetil (MMF). One was converted to scheduled rituximab redosing due to intolerance of both azathioprine and MMF. Six (9%) were not receiving maintenance immunosuppression due to intolerance or risk of infectious complications.

### Early response

**FIGURE 1:**
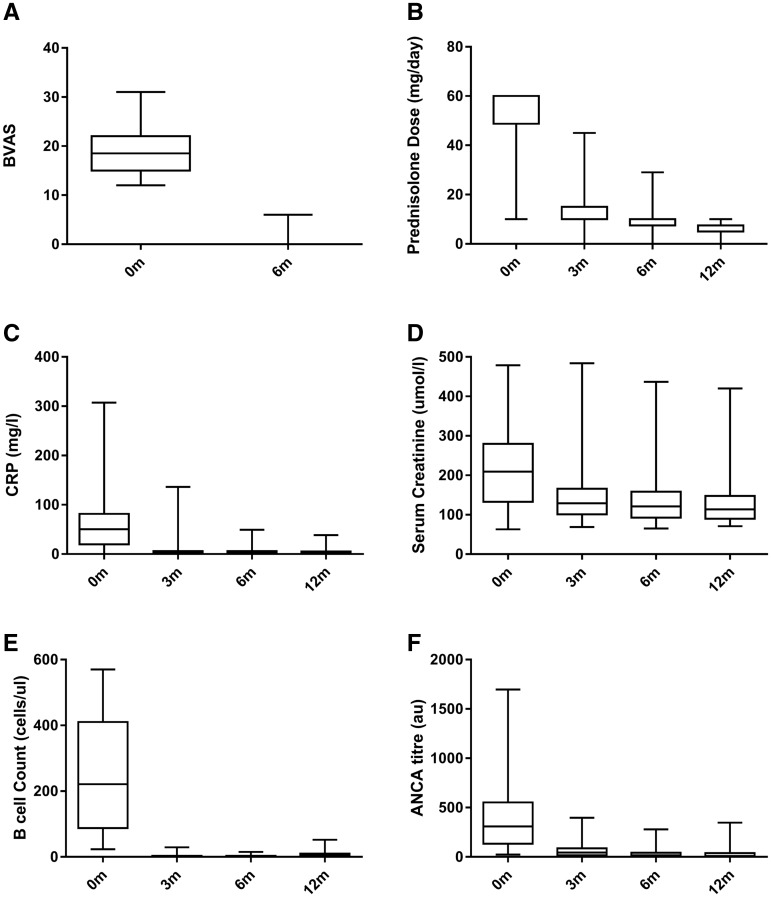
Early disease response at 6 and 12 months. (**A**) BVAS at 0 and 6 months. (**B**) Oral prednisolone dose (mg/day) during the first year of therapy. Fourteen patients received pulsed intravenous methylprednisolone prior to referral to our centre and in these cases the initial dose of oral prednisolone was reduced. (**C**) Sequential serum CRP (mg/L). **D** Serum creatinine (μmol/L). (**E**) Peripheral B lymphocyte count (cells/μL). (**F**) ANCA titres as determined by antigen specific assay (1F) at 3, 6 and 12 months. Box and whisker plots represent median (IQR) and range measurements.

Sixty-two patients (94%) achieved remission by 6 months (Figure 1A) and all but four were compliant with the protocolized corticosteroid taper at this time point (Figure 1B). In keeping with clinical response, there were rapid improvements in serum C-reactive protein (CRP; Figure 1C) and creatinine (Figure 1D) by Month 3, which were sustained during the first year of treatment. All patients achieved B-cell depletion (<10 cells/μL) within 3 months (Figure 1E) and this was associated with a rapid decrease in ANCA titres during the first year of treatment (Figure 1F).

Of the four patients who did not achieve remission by Month 6, one required the addition of plasma exchange and extended treatment with cyclophosphamide due to deteriorating renal function despite initial protocol treatment. One had persistent ENT symptoms and required additional oral corticosteroid therapy; she subsequently achieved remission by 9 months. Two patients died in the first 6 months; one due to a cerebrovascular accident at Month 1 and one due to multi-organ failure secondary to chest sepsis at Month 5.

### Long-term outcomes

**FIGURE 2:**
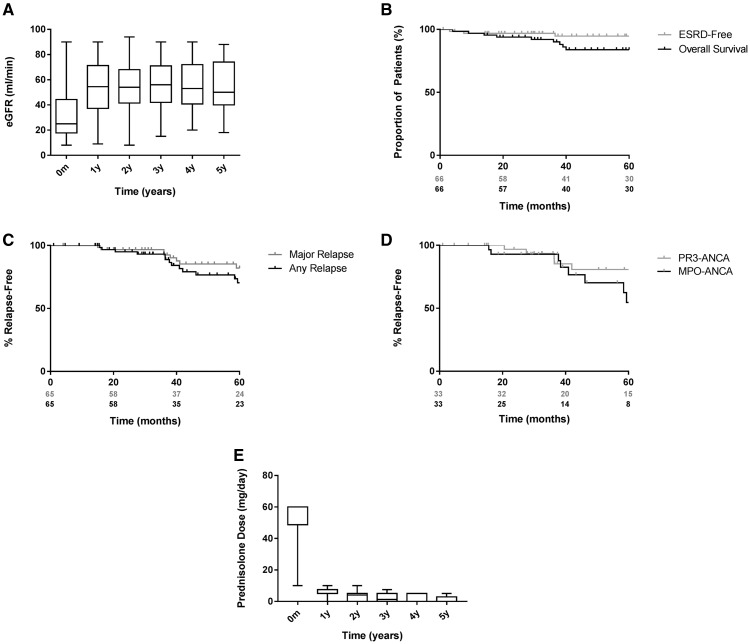
Long-term outcomes during 5-year follow-up. (**A**) eGRF (mL/min) at annual intervals during 5-year follow-up. Data censored at point of progression to end-stage renal disease for four patients. Unadjusted Kaplan–Meier survival functions describing (**B**) overall and ESRD-free survival (the latter censored for death) and relapse-free survival censored for death in (**C**) MPO-ANCA- and (**D**) PR3-ANCA-positive patients during the 5-year follow-up. (**E**) Long-term steroid exposure in the cohort during the 5-year follow-up (censored for relapse). Box and whisker plots represent median (IQR) and range measurements.

Figure 2A and B describe long-term changes in eGFR and renal survival, respectively. Four patients progressed to ESRD during follow-up (at 3, 8, 37 and 69 months). These patients tended to be older [median 71 years (range 58–82)] and had adverse indicators of renal outcome at presentation [median creatinine 352 μmol/L (range 213–479) and median tubular atrophy 25% (range 25–50)]. Three had mixed-class histology; the remaining patient had crescentic-class disease.

Fifty-seven patients were alive at the last follow-up (Figure 2B). Details of the nine deaths are summarized in Table 3. The median time to death after presentation was 29 months (range 1–67) and the median age at death was 77 years (range 57–88).

**Table 3:** Adverse events and deaths

Infectious adverse events			
At least one infection requiring i.v. antibiotics or admission			20
Zoster			5
CMV viraemia			2
Aspergillus			1
PJP			None
PML			None
Hepatitis reactivation			None
Non-infectious adverse events			
New diabetes			7
Osteoporosis or bone fracture			5
Venous thromboembolism			3
Cardiac events			3
Persistent hypo-IgG			4
Late-onset neutropenia			None
Cancer			6
SCC			2
BCC			1
Breast			1
Lung			1
Prostate			1
Deaths			
Case	Age (years)	Time point (months)	Cause
1	79	1	Cerebrovascular disease
2	70	5	Chest sepsis
3	57	15	Unknown
4	82	19	Unknown
5	63	29	Lung cancer
6	76	39	Sudden cardiac
7	88	39	Sudden cardiac
8	77	40	Chest sepsis (chronic airways disease)
9	86	67	Unknown

i.v., intravenous; CMV, cytomegalovirus; PJP, *Pneumocystis jiroveci* pneumonia; PML, progressive multifocal leucoencephalopathy; IgG, immunoglobulin G; SCC, squamous cell carcinoma; BCC, basal cell carcinoma.

Figure 2E describes the duration of steroid exposure during long-term follow-up. The median time to achieving corticosteroid dose ≤5 mg/day was 9.9 months and the median dose of oral corticosteroids at 1, 2 and 4 years was 5 mg (IQR 5–7.5), 4 mg (IQR 0–5) and 0 mg (IQR 0–5), respectively. At Years 2 and 4, 85 and 79% of surviving patients, respectively, continued on maintenance immunosuppressive.

### Relapse

At 1, 3 and 5 years, 100, 93 and 70% of surviving patients were free from relapse, respectively. Of the patients, 0, 8 and 15% had major relapse at these time points, respectively (Figure 2C). Of note, we did not observe a difference in relapse rate between MPO-ANCA- and PR3-ANCA-positive patients (P = 0.09; Figure 2D). At last follow-up, a total of 10 patients had experienced a major relapse. The majority of these (70%) occurred in patients who were positive for MPO-ANCA and all were ANCA positive at the time of relapse. Ninety percent had repopulated B cells (>10 cells/μL) and the majority (90%) were on maintenance treatment. The median time to major relapse was 39 months (range 15–75).

### Long-term B-cell and ANCA kinetics

**FIGURE 3:**
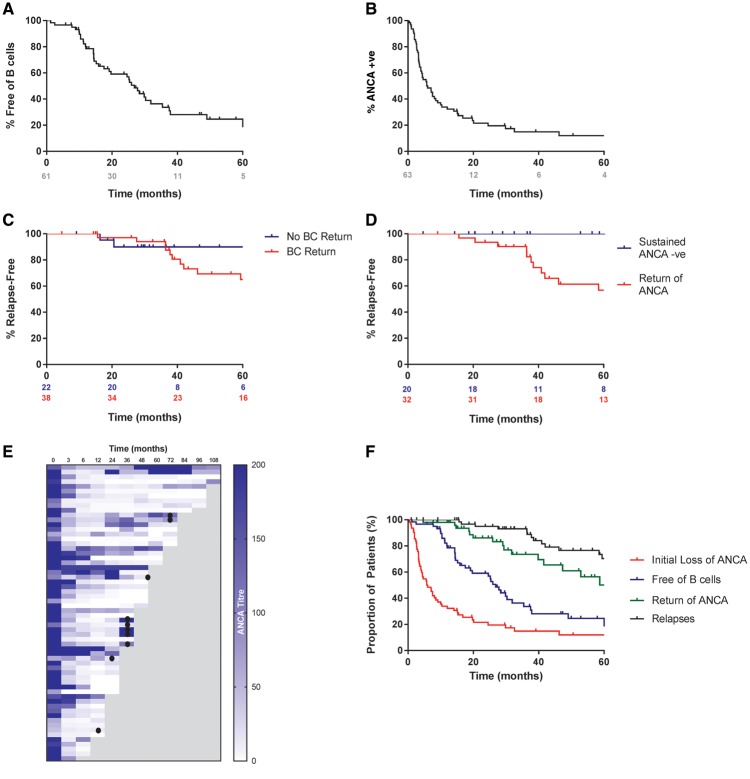
B-cell and ANCA kinetics and their relationship to relapse during the 5-year follow-up. Kaplan–Meier functions describing (**A**) the proportion of patients who remained free of peripheral B cells (<10 cells/uL) and (**B**) the time taken to achieve ANCA-negative testing as determined by antigen-specific assay during the 5-year follow-up. Kaplan–Meier functions describing the incidence of first major or minor relapse in (**C**) patients who had a return of peripheral B cells (>10 cells/uL) versus those who did not and (**D**) patients who remained ANCA negative versus those who had a return of ANCA in those patients who became ANCA negative after initial treatment during the 5-year follow-up. (**E**) Heatmap describing sequential ANCA measurements in the 62 patients who survived beyond 6 months and who were ANCA positive by antigen-specific assay during long-term follow-up. The occurrence of major relapse is indicated by filled circles. (**F**) Composite Kaplan–Meier curve describing the sequence of B-cell repopulation, return of circulating ANCA and the occurrence of relapse for the entire cohort over the 5-year follow-up. The red plot shows the time to achieve ANCA-negative testing, the blue plot describes the time for return of peripheral B cells, the grey plot describes the time for return of circulating ANCA in those patients who had previously achieved ANCA-negative testing and the black plot describes the time to the first minor or major relapse.

We observed prolonged B-cell depletion in this cohort. At 6, 12, 18 and 24 months, 97, 79, 63 and 57% of patients remained B-cell deplete (<10 cells/μL), respectively (Figure 3A). In total, 64% of patients had repopulated B cells at the last follow-up, with a median time to repopulation of 18 months (range 1–99) in those who did replete. ANCA-negative seroconversion occurred in 86% of patients (Figure 3B and E) at a median time of 4.4 months (range 0.4–46.3). Of those patients who achieved ANCA negativity, 60% had a subsequent return of ANCA at a median of 15.0 months (range 2.7–103.0). There was a trend for earlier return of MPO-ANCA versus PR3-ANCA (P = 0.07), which may have contributed to the unexpected similarity in relapse rates in these groups. Figures 2C and D describe the incidence of relapse in patients who had B-cell repopulation versus those who did not and in those who had sustained ANCA negativity versus those who did not, respectively. B-cell repletion and return of ANCA had a poor positive predictive value for relapse (28 and 38%, respectively) but they had a high negative predictive value (100 and 91%, respectively). Figure 2F describes the relationship of B-cell and ANCA kinetics to the incidence of relapse for the entire cohort.

### Adverse events

Table 3 details the adverse events seen during the entire follow-up. Twenty patients (30%) had at least one Grade III infection requiring intravenous antibiotic therapy or admission to hospital during a total median follow-up of 56 months. The overall serious infection rate during long-term follow-up was 1.24 per 10 patient-years. The infection rate tended to be lower in patients who repopulated B cells within 12 months (0.95 per 10 patient-years) compared with those who repopulated within or after 24 months (1.33 and 1.17 per 10 patient-years, respectively). Four patients developed persistent hypogammaglobulinaemia, two of whom required treatment with intravenous immunoglobulin. We did not observe late-onset neutropenia. A small number of non-skin cancers (*n* = 3) and non-melanoma skin cancers (*n* = 3) were detected.

### Case–control study with EUVAS trial patients

**FIGURE 4:**
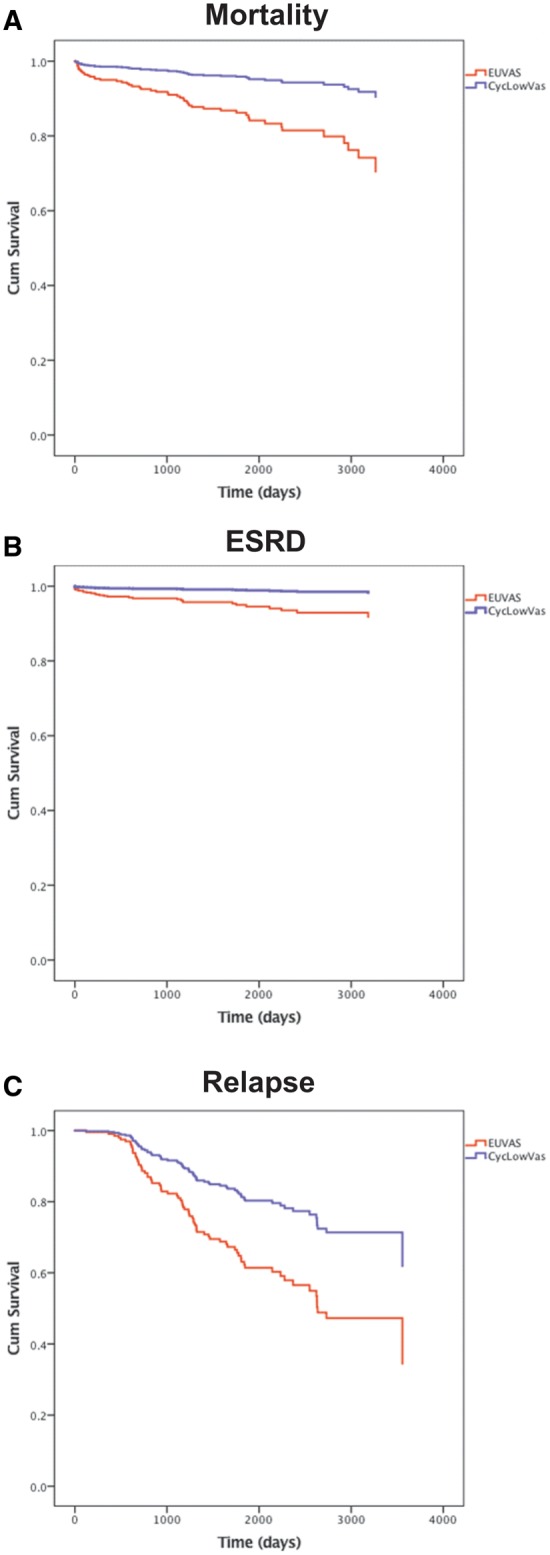
Case–control analysis with propensity-matched patients from EUVAS trials**.** Cox proportional hazards regression models. (**A**) The adjusted all-cause mortality, (**B**) ESRD and (**C**) relapse risk between the EUVAS cohort and combined treatment (‘CycLowVas’) cohort. The covariates in the analysis include age, sex, ANCA specificity, entry eGFR, entry BVAS and dose of cyclophosphamide.

Table 4 describes the baseline features of our cohort (‘CycLowVas’) compared with propensity-matched control patients enrolled in previous EUVAS studies. The patients were well matched for age, ANCA specificity, BVAS and CKD stage at presentation. Rates of remission and changes in eGFR at 6 months were comparable between groups and, in unadjusted analyses, the proportion of patients encountering relapse was not statistically different for the two patient cohorts (21% versus 30.8%; P = 0.18). However, PR3-ANCA-positive patients treated with the CycLowVas regimen were significantly less likely to relapse (15% versus 43%; P = 0.008). In multivariable analyses, treatment with the CycLowVas regimen was associated with a reduced risk of death {HR 0.29 [95% confidence interval (CI) 0.125–0.675], P = 0.004}, progression to ESRD [HR 0.20 (95% CI 0.06–0.65), P = 0.007] and relapse [HR 0.49 (95% CI 0.25–0.97), P = 0.04]. Figure 4 shows the Cox proportional hazards regression curves describing the long-term risk of death, ESRD and relapse.

**Table 4:** Case–control comparison with EUVAS trials

	CycLowVas cases	EUVAS controls	P-value
*n*	66	198	
Baseline features			
Female:male, *n* (%)	28:38 (42:58)	78:120 (40:60)	0.66
Age (years) median (range)	63 (54.2–73)	63 (53.25–71)	0.75
MPO:PR3 ANCA, *n* (%)	33:33 (50:50)	99:99 (50:50)	1.00
Baseline eGFR (mL/min), median (range)	25.5 (18–43.7)	27.1 (16.2–40.6)	0.45
Baseline BVAS, median (range)	18.5 (15–21.75)	18.5 (13–23.75)	0.77
Unadjusted outcomes at 6 months			
Remission at 6 months, *n* (%)	62/66 (96)	141/151 (93.7)	0.88
6-month eGFR (mL/min), median (range)	49 (37.5–67)	45.7 (31.8–62.23)	0.14
6-month ΔeGFR (mL/min), median (range)	13 (1.5–27.5)	11.3 (1.5–24.15)	0.30
Cyclophosphamide dose (g), median (range)	3 (3–3.5)	13.5 (8.6–22.5)	<0.001
Total corticosteroid dose (g), median (range)	4.2 (1.0–6.5)	5.3 (1.1–9.3)	<0.001
Unadjusted long-term outcomes, *n* (%)			
Relapse	14/66 (21)	61/198 (30.8)	0.18
Relapse PR3-ANCA	5/33 (15)	42/99 (42)	0.008
Relapse MPO-ANCA	9/33 (27)	19/99 (19)	0.46
ESRD	4/66 (6)	28/198 (14)	0.12
Death	9/66 (13.6)	46/198 (23.2)	0.13
Follow-up (days) median (range)	1716 (964–2598)	1878 (999–2946)	

## DISCUSSION

This combined treatment regimen provided early disease control in renal AAV and was effective in 94% of patients at 6 months. Long-term patient and renal survival were favourable and relapse rates were lower than expected compared with previously published cohorts treated with purely cyclophosphamide-based regimens.

There are limited data describing the use of rituximab-based therapy in patients with severe renal involvement in AAV. Small case series suggest that rituximab may be effective in this context, although cases were heterogeneous [19, 20]. In the largest controlled study to date, the RAVE trial, only 50% of patients had renal involvement. While *post hoc* analysis suggested that outcomes of patients with renal involvement were comparable in the subgroups treated with rituximab and cyclophosphamide, it is notable that excretory renal function at entry was relatively well preserved in these patients, with eGFRs in the range of 40–60 mL/min [21]. Although patients in the RITUXVAS study had more severe renal involvement, with an average eGFR of 18 mL/min, patients in the rituximab treatment arm also received two intravenous doses of cyclophosphamide, with the stated aim of ensuring early disease control and to prevent the potential induction of human anti-chimeric antibodies (EUDRACT 2005-003610-15) [5]. It has been suggested that cyclophosphamide may have effects beyond B-cell depletion that are of early benefit in renal disease, such as elimination of pathogenic T cells or intrarenal macrophages [22]. We agree with this rationale and believe that cyclophosphamide may be a beneficial component of initial treatment for patients with renal impairment secondary to severe pauci-immune glomerulonephritis to ensure rapid suppression of renal inflammation and prevention of long-term damage.

Previous studies have shown that an increased dose or longer duration of cyclophosphamide treatment is associated with lower rates of relapse [23, 24], with the corollary of increased rates of adverse events such as infection and malignancy [25–28]. Our experience suggests that the addition of 2 × 1 g doses of rituximab to the induction regimen permits reduction in the cyclophosphamide dose required to achieve prolonged remission. Our patients received 3 g cyclophosphamide, compared with 8.2 and 15.9 g in the pulsed intravenous and daily oral arms, respectively, of the CYCLOPS study [29], and showed sustained disease-free remission, with relapse rates that were comparable between PR3- and MPO-ANCA-positive cases. These observations are in keeping with other findings that suggest rituximab may be particularly effective in preventing relapsing disease and in those who are PR3-ANCA positive. In the RAVE study, PR3-ANCA-positive patients had better initial response to rituximab versus cyclophosphamide [30]. In addition, subgroup analysis found that rituximab was particularly beneficial in patients who were treated for relapsing disease, and longer term follow-up implied that a single treatment with rituximab was non-inferior to conventional continuous oral immunosuppression for prevention of relapse at 18 months [31].

We observed prolonged peripheral B-cell depletion in our cohort, which we believe contributed to the reduced risk of relapse and improved long-term outcomes. At 2 years, 50% of patients remained B-cell deplete without further cytotoxic therapy. This is in contrast to the RAVE and RITUXVAS studies, in which the majority of rituximab-treated patients reconstituted B by 12 months [31, 32], suggesting a synergistic effect whereby cyclophosphamide and rituximab potentiate their respective effects on B-cell elimination. Anti-CD20 monoclonal antibody treatment has been shown to sensitize lymphoma cell lines to cyclophosphamide-mediated cytotoxicity *in vitro* [33], and multitarget therapy is a well-established strategy in the oncology clinic. Sustained B-cell depletion with repeated rituximab treatment has been associated with a decreased risk of relapse in other studies [6, 7], and our experience suggests that this may effectively be achieved with combination therapy at induction.

B-cell depletion was associated with high rates of seroconversion to ANCA negativity (86%), which occurred in the first 6 months of therapy for most patients, an observation that has been associated with a reduced relapse risk in other cohorts [34]. It is striking that, as a cohort, B-cell repopulation preceded the recurrence of ANCA, which in turn preceded the development of relapse (Figure 3F), and that 90% of major relapses occurred in patients who were both B-cell replete and ANCA positive. This is in keeping with recent evidence that ANCA may be a useful predictor of relapse in patients with renal involvement [35]; however, because the overall relapse rate was low, we were unable to identify B-cell or ANCA parameters that were positively predictive of recurrence. Reassuringly, prolonged B-cell depletion was not associated with an unexpectedly high incidence of adverse events. Rates of infection and hypogammaglobulinamia were comparable with other studies, including a recent analysis of patients multiply re-treated with rituximab [11]. Nor did we observe an excess of malignancies, in keeping with a recent retrospective analysis that suggested patients with AAV treated with rituximab have a comparable risk of malignancy with the general population [36].

While this protocol used combination cytotoxic therapy, it was relatively corticosteroid sparing. Patients did not receive intravenous corticosteroids at our centre and oral corticosteroids were rapidly tapered during the first month, giving a median total dose of 4.2 g by 6 months. This is in contrast to the CYCLOPS study, where an average corticosteroid dose of 7.5 g was administered during induction, and the RAVE study, where high-dose intravenous corticosteroids were employed and initial doses of oral prednisolone of 40–60 mg were maintained for the first month of therapy (giving a cumulative total oral and intravenous dose approaching 6 g). Similarly, our regimen used less cumulative corticosteroids than most reported studies of induction therapy in AAV (Supplementary data, Table S1 and reference [37]). It is possible that the avoidance of high-dose corticosteroid exposure, and the associated toxicity [38, 39], may have allowed the safe combination of two cytotoxic therapies without encountering an excess of infectious complications. Curtailment of corticosteroid therapy has been associated with an increased risk of relapse using conventional treatment regimens [40], although this risk may likewise be outweighed by the benefit accrued from combination cytotoxic therapy for long-term disease control, and we suggest that this combined regimen may provide the basis for early corticosteroid withdrawal in AAV. Results from the ongoing PEXIVAS (Plasma Exchange and Glucocorticoids for Treatment of Anti-Neutrophil Cytoplasm Antibody-Associated Vasculitis) study, which includes randomization to either standard (estimated 4.2 g) or reduced dose (2.3 g) oral corticosteroids, may provide further information on the efficacy and safety of such an approach [41].

A limitation of our case–control analysis is the use of a historical group of patients enrolled to EUVAS studies during a time period that predates our cohort’s recruitment. It is recognized that survival in AAV has improved over recent decades [42–44], such that factors other than drug protocol may have contributed to the improved outcomes in our more recently treated cohort. It is notable, however, that the largest of these studies did not observe a change in relapse frequency over time [44], indicating that the lower relapse rate observed in our cohort may remain significant. A further limitation of our study is that the use of maintenance therapy was not standardized and this may, along with the combination induction regimen, have contributed to the long-term outcomes we described. The recently published findings of the EUVAS coordinated REMAIN (Randomised Trial of Prolonged Remission-Maintenance Therapy in Systemic Vasculitis) study for example confirm that prolonged maintenance treatment is associated with reduced risk of relapse [45], although the applicability of its results in the rituximab-based induction era may be limited. In addition, we have not examined the efficacy of this regimen in patients presenting with severe disease manifestations requiring plasma exchange, such as those presenting with renal failure requiring dialysis or diffuse alveolar haemorrhage, and our cohort included a low proportion of patients with relapsing disease at enrolment. Finally, as a clinical cohort study, we were unable to examine the B-cell phenotype and kinetics with the granularity described in recent mechanistic work, and it would be of interest to define these features in more detail in the future [46, 47].

## CONCLUSION

This report has the intrinsic limitations of a single-centre, uncontrolled, open-label study, although its strengths include a highly standardized cytotoxic treatment regimen, long-term follow-up and a formal case–control comparison with published trials suggesting favourable outcomes. We propose that prospective studies are desirable to compare the use of this regimen, and other B-cell-depleting strategies [48], with current standard treatment protocols (and in particular to purely rituximab-based induction and maintenance strategies and in patients with relapsing disease) and to establish if this regimen may provide the basis for further corticosteroid avoidance in the management of renal AAV.

## Supplementary Material

Supplementary Table S1
