# Supplementary material for: Long-term follow-up of a combined rituximab and cyclophosphamide regimen in renal anti-neutrophil cytoplasm antibody-associated vasculitis
Source: Nephrol Dial Transplant. 2018 Feb 14;34(1):63–73. doi: 10.1093/ndt/gfx378 (PMC6322443; doi:10.1093/ndt/gfx378)
Supplement: Supplementary Table S1 [file gfx378_supplementary_table_1.docx]

| **Supplementary Table 1: Corticosteroid Regimens in Previous and Ongoing Studies of Remission-induction Treatment** | | |
| --- | --- | --- |
| **Study** | **i.v. Methylprednisolone (g)** | **Calculated Oral**  **Prednisolone at six months^1^ (g)** |
| CYCLOWVAS | 0 | 3.0 |
| NORAM | 0 | 3.6 |
| CYCLOPS | <1 | 3.4 |
| RITUXIVAS | 1 | 3.4 |
| RAVE | 1-3 | 3.5 |
| PEXIVAS – Standard dose | 1-3 | 4.0 |
| PEXIVAS – Reduced dose | 1-3 | 2.3 |
| ^1^Based on body weight 60kg | | |
